# Patient symptom experience prior to a diagnosis of oesophageal or gastric cancer: a multi-methods study

**DOI:** 10.3399/bjgpopen20X101001

**Published:** 2020-01-08

**Authors:** Elka Humphrys, Fiona M Walter, Greg Rubin, Jon D Emery, Margaret Johnson, Anthony Richards, Rebecca C Fitzgerald, Yirupaiahgari KS Viswanath, Jenni Burt

**Affiliations:** 1 Research Associate, The Healthcare Improvement Studies Institute, University of Cambridge, Cambridge, UK; 2 Principal Researcher in Primary Care Cancer Research, Department of Public Health and Primary Care, University of Cambridge, Cambridge, UK; 3 Emeritus Professor of General Practice and Primary Care, Institute of Health and Society, Newcastle University, Newcastle-upon-Tyne, UK; 4 Herman Professor of Primary Care Cancer Research, Department of General Practice and Primary Health Care, Centre for Cancer Research, University of Melbourne, Melbourne, Australia; 5 Lay Member, Department of Public Health and Primary Care, University of Cambridge, Cambridge, UK; 6 Lay Member, Department of Public Health and Primary Care, University of Cambridge, Cambridge, UK; 7 Professor of Cancer Prevention, MRC Cancer Unit, University of Cambridge, Cambridge, UK; 8 Professor of Surgery, James Cook University Hospital, South Tees Hospitals NHS Foundation Trust, Middlesbrough, UK; 9 Senior Social Scientist, The Healthcare Improvement Studies Institute, University of Cambridge, Cambridge, UK

**Keywords:** Primary health care, gastrointestinal neoplasms, early diagnosis, interviews, surveys and questionnaires, esophageal neoplasms, general practice

## Abstract

**Background:**

Late stage diagnosis of oesophageal and gastric cancer is common, which limits treatment options and contributes to poor survival.

**Aim:**

To explore patients' understanding, experience and presentation of symptoms before a diagnosis of oesophageal or gastric cancer.

**Design & setting:**

Between May 2016 and October 2017, all patients newly diagnosed with oesophageal or gastric cancer were identified at weekly multidisciplinary team meetings at two large hospitals in England. A total of 321 patients were invited to participate in a survey and secondary care medical record review; 127 (40%) participants responded (102 patients had oesophageal cancer and 25 had gastric cancer). Of these, 26 participated in an additional face-to-face interview.

**Method:**

Survey and medical record data were analysed descriptively. Interviews were analysed using thematic analysis, informed by the Model of Pathways to Treatment.

**Results:**

Participants experienced multiple symptoms before diagnosis. The most common symptom associated with oesophageal cancer was dysphagia (*n* = 66, 65%); for gastric cancer, fatigue or tiredness (*n* = 20, 80%) was the most common symptom. Understanding of heartburn, reflux and indigestion, and associated symptoms differed between participants and often contrasted with clinical perspectives. Bodily changes attributed to personal and/or lifestyle factors were self-managed, with presentation to primary care prompted when symptoms persisted, worsened, or impacted daily life, or were notably severe or unusual. Participants rarely presented all symptoms at the initial consultation.

**Conclusion:**

The patient interval may be lengthened by misinterpretation of key terms, such as heartburn, or misattribution or non-recognition of important bodily changes. Clearly defined symptom awareness messages may encourage earlier help-seeking, while eliciting symptom experience and meanings in primary care consultations could prompt earlier referral and diagnosis.

## How this fits in

Approximately 70% of oesophageal and gastric cancers are diagnosed at a late stage, contributing to poor survival outcomes observed for these cancers. There is limited evidence exploring the pathways to diagnosis from the patient’s perspective; this study highlights the multiple symptoms typically experienced before help-seeking in primary care. Some differences were identified in symptom experience between patients newly diagnosed with oesophageal or gastric cancer, and there was variation in patient understanding of key terms such as heartburn, reflux and indigestion, and the symptoms associated with these conditions. The results highlight the importance of exploring symptom understanding and experience with patients in primary care to avoid missed opportunities for timely diagnosis.

## Introduction

Oesophageal and gastric cancers, often referred to as oesophago-gastric cancers, account for 5% of all new cancer cases in the UK, with over 15 000 cases diagnosed in 2015.^1,2^ Approximately 70% are diagnosed at a late stage (III or IV),^1,2^ and less than 20% of patients are alive after 5 years.^3,4^ An analysis of cancer diagnosis audit data identified that patients with oesophageal cancer experienced a median patient interval, the time between symptom onset and presentation in primary care, of 21.5 days (interquartile range [IQR] 7–46; gastric median 9 days, IQR 0–38), while patients with gastric cancer experienced a longer primary care interval, from presentation to referral (median 12 days, IQR 0–65; oesophageal median 5 days, IQR 0–30).^5^

Motivated by a policy commitment to achieve earlier cancer diagnoses and reduce mortality,^6^ the 2015 Be Clear on Cancer campaign highlighted potential oesophago-gastric cancer symptoms, encouraging people to seek medical advice for recurrent heartburn or difficulty swallowing (dysphagia).^7^ Although evidence suggests that heartburn is associated with earlier stage oesophageal cancer,^8^ patients may not understand the term ‘heartburn’^9^ and, while urgent referral from primary care is recommended for all patients presenting with dysphagia,^10^ this symptom is often associated with later stage disease.^11,12^ An evaluation of the pilot study in the north of England demonstrated a significant increase in cancers diagnosed in those aged 60–69 years^13^ during the campaign period; however, these findings were not replicated in the national campaign.^14,15^

While dysphagia is considered an ‘alarm symptom’, prompting urgent referral for suspected cancer,^10^ other symptoms presented to primary care can be similar to benign gastrointestinal disorders.^16^ Clinician symptom assessment does not always match patients’^17^ and misattribution of symptoms could be contributing to up to a third of patients requiring three or more primary care consultations before referral.^18^

There is little patient-reported data on how people appraise and seek help for possible symptoms of oesophageal or gastric cancer, to underpin efforts to encourage earlier diagnosis. The aim of this study was, therefore, to explore patient’s understanding, experience, and presentation of symptoms before diagnosis.

## Method

A multi-method approach was used, which comprised a patient survey, secondary care case-note review, and semi-structured patient interviews.

### Recruitment

Eligible patients (aged ≥18 years, newly diagnosed with oesophageal or gastric cancer) were identified at weekly regional upper gastrointestinal multidisciplinary team meetings at two large hospitals in the east and north east of England from May 2016–October 2017. Research or specialist nurses approached eligible patients within 4 weeks of diagnosis where possible, through face-to-face or postal contact. Patients received an information sheet, study questionnaire, consent form, and freepost envelope to return completed documents to the study team, with the option to participate by phone. Patients assessed as ineligible by their diagnosing clinician (for example, serious comorbidities, unable to communicate in English) were excluded. Non-responders were not sent reminders.

Participants willing to be interviewed were purposively sampled by age, sex, cancer type, and region (east or north east) for a face-to-face interview conducted within 10 weeks of diagnosis (June 2016–July 2017) at a location chosen by the participant. Written informed consent was obtained before participation.

### Data collection

The study questionnaire used previously validated items from the SYMPTOM UK cohort studies^19–21^ to gather data on symptom onset and first presentation to the GP (free-text response and dates). An item from the 2014 National Cancer Patient Experience Survey^22^ determined the number of pre-referral GP consultations. Newly developed items explored medication use before diagnosis, along with symptom experience, and frequency (all or most of the time, some of the time, rarely, or never) based on a list of 12 symptoms (Box 1A). Items on education, living arrangements, ethnic group, sex, and comorbidities were also included. The questionnaire was assessed for face validity and usability in a think-aloud study^23,24^ with eight participants, with minor modifications made before use in the main study.

Box 1 **Questionnaire and interview items**

**A: Symptoms explored in the questionnaire**

- Burning feeling behind your breastbone (heartburn)
- Stomach contents moving upwards to your throat or mouth (regurgitation)
- Liquid or food sticking when you swallow (dysphagia)
- Pain when you swallow (pain swallowing)
- Pain or tenderness in the upper part of your stomach (upper abdominal pain)
- Fullness or bloating in the upper part of your stomach (fullness or bloating)
- Decrease in appetite (decreased appetite)
- Nausea
- Vomiting
- Vomiting blood
- Unexplained weight loss (weight loss)
- Fatigue or tiredness

**B: Key areas of the interview topic guide**

1. Introduction — study overview, data protection and confidentiality, consent, any questions
2. Opening question — encourage participants to talk about themselves, explore daily life prior to the symptoms or diagnosis, familiarise with participant’s social network
3. Symptom experience — exploration of bodily changes and symptom experiences, timeline of events, appraisal of symptoms, prior knowledge, information-seeking, symptom management, social influence
4. Healthcare experience — help-seeking, exploration of first consultation, subsequent consultations, timeline of events, relationship with healthcare provider, opinions about the consultation(s), thoughts and feelings about the diagnosis
5. Interview close — confirmation of data protection and confidentiality, any questions, thanks

( ) Symptom abbreviation for reporting purpose

Interviews were conducted by one researcher, with experience of qualitative research, using a semi-structured topic guide (Box 1B) developed for the study and informed by previous qualitative studies.^25–27^ A calendar landmarking approach^28,29^ aided event recall. All interviews were audio-recorded (duration 42–111 minutes) and transcribed verbatim. Memos were used to reflect on interviews.

Secondary care medical records confirmed each participant’s pre-treatment disease stage and histological diagnosis (date and findings).

### Analysis

Survey data were imported to IBM SPSS (version 25) and analysed descriptively to explore participant characteristics (cancer type, age, sex, ethnic group, and region), comorbidities, symptoms (total cohort and by cancer type), and healthcare experience before diagnosis (over-the-counter medication, reported GP consultations, and referral route), and pre-treatment disease stage. The time between first noticing and first reporting a symptom to the GP was calculated using participant-reported exact or estimated dates (2-year timeframe), with analysis rules from previous studies used to convert the latter to estimated exact dates.^19–21^ Pearson’s χ^2^ test was used to evaluate non-response based on age and sex.

Interview audio files were professionally transcribed and transcripts uploaded to NVivo (version 11) for data management. A thematic analysis approach^30^ was used to code the dataset and identify themes, with the Model of Pathways to Treatment^31,32^ providing a framework to understand the pathways to diagnosis, as recommended by the Aarhus Statement.^33^ One researcher read and coded all transcripts as the interviews progressed, comparing experiences across the whole cohort and between cancers. A subset of transcripts were reviewed by four members of the research team, with final themes discussed and agreed throughout the analysis.

## Results

Between May 2016 and October 2017, newly diagnosed patients were discussed at weekly regional multidisciplinary team meetings, with eligible patients (*n* = 321) identified and approached by the two recruiting hospitals. A total of 127 patients completed the questionnaire (40% recruitment rate), the majority (*n* = 109, 86%) within 6 weeks of diagnosis. Participants included 102 with oesophageal cancer and 25 with gastric cancer. Twenty-seven participants were invited for interview, and 26 interviews were completed (one non-responder to invite), all within 10 weeks of diagnosis. Participant, healthcare, and disease characteristics are presented in Table 1.

**Table 1.** Participant, healthcare, and disease characteristics

	Survey participants(*n* = 127)^a^	Interview participants(*n* = 26)^a^
**Age, years**		
Median (range)	71 (44–96)	69.5 (55–88)
<60	12 (9.4^b^)	3 (11.5)
60–74	78 (61.4^b^)	12 (46.2)
≥75	37 (29.1^b^)	11 (42.3)
**Sex**		
Male	102 (80.3)	18 (69.2)
Female	25 (19.7)	8 (30.8)
**Cancer type**		
Oesophageal^c^	102 (80.3)	15 (57.7)
Gastric^d^	25 (19.7)	11 (42.3)
**Ethnic group**		
White	124 (97.6)	26 (100.0)
Other	3 (2.4)	0 (0.0)
**Region**		
East	77 (60.6)	13 (50.0)
North East	50 (39.4)	13 (50.0)
**Comorbidities**		
Arthritis	38 (29.9)	13 (50.0)
Cancer — other than O/G	22 (17.3)	5 (19.2)
Heart disease	17 (13.4)	2 (7.7)
Lung disease	13 (10.2)	2 (7.7)
Anxiety or depression	13 (10.2)	2 (7.7)
Barrett’s or reflux disease	10 (7.9)	1 (3.8)
Any of the above	72 (56.7)	16 (61.5)
**OTC medication**		
None	79 (62.2)	17 (65.4)
Antacid or similar	30 (23.6)	7 (26.9)
Other	8 (6.3)	1 (3.8)
**Reported GP consultations**		
None^e^	13 (10.2)	6 (23.1^b^)
1	56 (44.1)	10 (38.5^b^)
2	35 (27.6)	4 (15.4^b^)
≥3	21 (16.5)	6 (23.1^b^)
**Referral route**		
Urgent^f^	88 (69.3)	16 (61.5)
Routine	19 (15.0)	6 (23.1)
Other	9 (7.1)	2 (7.7)
		
**Urgent referral based on GP consultations**		
None^e^	8 (6.3)	2 (7.7^b)^
1	41 (32.3)	8 (30.8^b)^
2	24 (18.9)	2 (7.7^b)^
≥3	15 (11.8)	4 (15.4^b)^
**Cancer stage pre-treatment**		
I/II	59 (46.5)	12 (46.2)
III/IV	64 (50.4)	14 (53.8)

^a^Values are *n* (%) unless otherwise stated. Missing data not included ^b^Percentages subject to rounding error. ^c^Tumours located in the upper or middle or lower oesophagus and gastro-oesophageal junction, including both adenocarcinomas and squamous cell carcinomas. ^d^Tumours located in the cardia, fundus, body, antrum, or pylorus. ^e^Patient referred opportunistically, that is, having attended primary care for another reason. ^f^Urgent referral is the 2-week wait suspected cancer pathway.

O/G = oesophageal or gastric. OTC = over the counter.

Comparing survey responders with non-responders, age was significantly associated with participation (Pearson’s χ^2^ = 8.043, *P* = 0.018), with fewer participants aged <60 years (9.3% versus 18.5%) and more aged 60–74 years (61.2% versus 46.6%). There was no difference based on sex.

Study data were used to explore participants’ knowledge of oesophageal and gastric cancer, understanding of heartburn (a key symptom in awareness campaigns), appraisal of symptoms, and help-seeking. In the discussion of each theme, survey and interview data are presented using a narrative synthesis approach,^34^ with a textual account of relevant findings from both data sources. Interview quotations are presented verbatim and contextualised with the participant ID, sex, age group (<60 years, 60–74 years, or ≥75 years), and cancer type.

### Knowledge of oesophageal and gastric cancer

Participants reflected on their limited knowledge of oesophageal and gastric cancer and the associated symptoms: *'I didn’t know much about stomach cancer'* (P22: male, aged 60–74 years, gastric)*; 'I’ve never heard of it, I’ve never heard of the oesophagus even'* (P01: male, aged <60 years, oesophageal). One participant recalled hearing the radio advertisement for the Be Clear on Cancer campaign,^7^ which highlighted dysphagia, while another participant read about the symptom in a magazine. Despite experiencing this symptom, these participants initially did not act on the advice to seek help:

*'* *I heard the advert and I took no notice of it.* *'* (P04: male, aged <60 years, oesophageal)

*'* *I’d read it in a magazine* [*…*] *I thought,* *"* *Oh yes, a lot of that relates to me* *"* *but still you think,* *"* *No* [*…*] *carry on, you’re fine* *"* *you know, which I’ve done for the last* *18* *months* *.*' (P03: female, aged 60–74 years, oesophageal)

Based on symptoms reported in the questionnaire (Box 1A), 74 (58%) participants experienced dysphagia before diagnosis, although this was primarily participants with oesophageal cancer (*n* = 66, 65%) compared with gastric cancer (*n* = 8, 32%). Fifty-two (41%) participants experienced heartburn, a major symptom of the Be Clear on Cancer campaign (*‘* *heartburn most days for three weeks or more’*).^7^ Of these participants, most reported heartburn some of the time (*n* = 41, 79%), with only 11 (21%) participants experiencing it more frequently (all or most of the time).

#### Understanding heartburn

When asked about heartburn experience, many participants described it as a burning sensation or acid taste, while some referred to mild pain:

*'* *I know what heartburn is and it’s a burning sensation* *.'* (P18: male, aged ≥75 years, oesophageal)

*'*[Heartburn] *is just this, I don’t know, rotten taste really* [*…*] *it’s acid.* *'* (P26: female, aged ≥75 years, gastric)

*'* *Mild pain to the right of my chest, which is traditionally the heartburn area, as I understand it.* *'* (P17: male, aged 60–74 years, oesophageal)

These symptoms were also ascribed to reflux and indigestion — *'it really does have that kind of burning feel about it when I had reflux'* (P06: male, aged <60 years, oesophageal) — with some participants using ‘heartburn’, ‘reflux’, and ‘indigestion’ interchangeably: *'I get discomfort … with the indigestion, and heartburn, and my tummy sometimes used to feel … a bit sore'* (P22: male, aged 60–74 years, gastric). However, some participants understood reflux and indigestion in relation to other symptoms, such as burping and regurgitation, along with severe pain associated with indigestion, suggesting the meanings of these terms may differ between participants:

*'* *It started with reflux* [*…*] *so a bit like a burp down here and eventually that translates into going to the toilet and having to be sick.* *'* (P18: male, aged ≥75 years, oesophageal)

*'*[Reflux] *was very definitely something that was sort of coming back up* [*…*] *into my throat.* *'* (P05: female, aged 60–74 years, gastric)

*'* *It did feel like indigestion* [*…*] *but was quite painful and seemed to spread* [*…*] *it was actually causing some pain in my head as well.* *'* (P06: male, aged<60 years, oesophageal)

**Figure 1.**
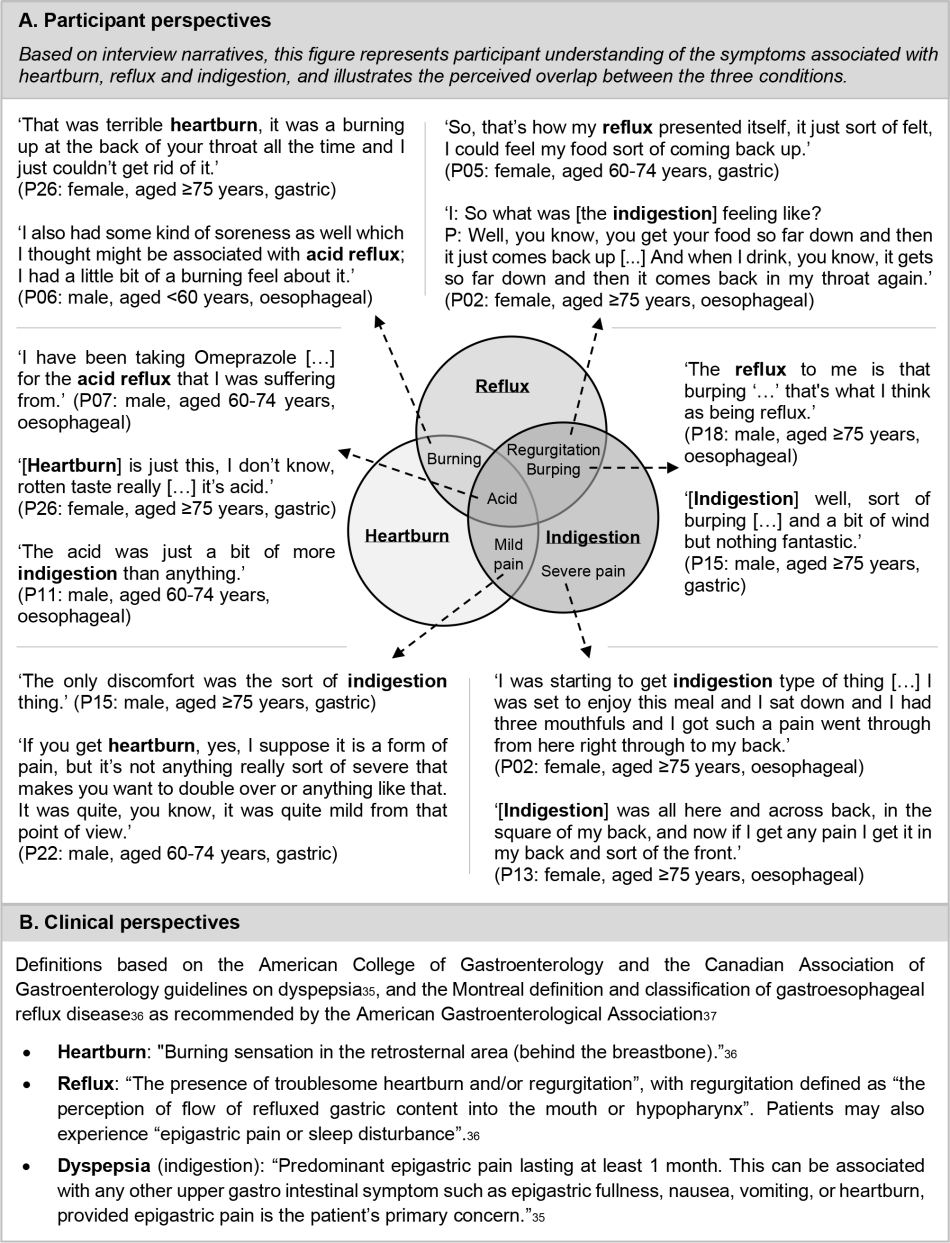
Participant and clinical perspectives of heartburn, reflux, and indigestion.^35–37^

One participant described how he had *'a couple of issues with regurgitation'* (P25: male, aged 60–74 years, gastric)*,* yet did not attribute this to reflux as he believed that heartburn and indigestion were the symptoms of reflux: *'I wasn’t getting the general symptom of acid reflux, which is indigestion and heartburn.*' Figure 1A illustrates participants’ understanding of symptoms and the perceived overlap between the conditions, in comparison with clinical perspectives presented in Figure 1B.

Based on questionnaire reported symptom experience, almost half of the total cohort (*n* = 62, 49%) reported regurgitation, while 45 (35%) participants experienced upper abdominal pain before diagnosis. When combining participant experience of these symptoms with those reporting a burning sensation, 89 (70%) participants experienced at least one of these symptoms.

### Symptom appraisal

Most participants were symptomatic, with 116 (91%) participants experiencing at least one symptom, and 110 (87%) reporting multiple symptoms before diagnosis. There were variations in the most commonly reported symptoms between cancers, with fatigue or tiredness the most common symptom experienced by participants with gastric cancer (*n* = 20, 80%), and dysphagia the most common for oesophageal participants (*n* = 66, 65%).

Symptoms were often normalised in the early stages of appraisal, with participants unconcerned by initial changes, attributing them to other factors including age, work, and lifestyle:

*'* *You’re* [*…*] *over 60 and you’re doing a job like that, and you’re thinking,* *"* *Well, I’m getting older now, so I’m bound to be feeling a bit more tired* *".'* (P22: male, aged 60–74 years, gastric)

*'* *I just noticed that I was having trouble getting food to go down my oesophagus* [*…*] *I was doing the* *5* *+* *2* *dieting so I thought maybe it was some peculiar effect.* *'* (P06: male, aged <60 years, oesophageal)

Heartburn, reflux, and indigestion were also viewed as a normal part of everyday life, with this view reinforced by advertisements for antacids, promoting over-the-counter medication to manage symptoms:

*'* *A bit of indigestion, if you will, nothing drastic, more indigestion than anything, and I didn’t bother about it, no reason.* *'* (P11: male, aged 60–74 years, oesophageal)

*'* *You'd say to somebody, “I've got acid reflux* *“* [...] *everybody in the world has acid reflux.* *'* (P18: male, aged ≥75 years, oesophageal)

*'* *You see the adverts on the television for Gaviscon and Rennies* [*…*] *you get the people with the brushes swishing out the inside of the stomach* [*…*] *and that very satisfied look on a person’s face, and that’s what you think heartburn is all about.* *'* (P17: male, aged 60–74 years, oesophageal)

Thirty-eight (30%) participants reported using over-the-counter medication to manage symptoms, primarily antacids or similar (*n* = 30, 24%), with interview participants describing their use in attempting to ease indigestion. Some were successful in initially managing their symptoms, while others found these medications ineffective:

*'* *I was trying to cure it myself like with Gaviscon and other patent medicines which didn’t work* [*…*] *I’d swallow it and it’d just come straight back up again.* *'* (P08: male, aged ≥75 years, oesophageal)

*'* *I bought the double tablets for them and when I was going down the road for a walk I used to shove a couple in my pocket just in case indigestion came on.* *'* (P23: female, aged ≥75 years, gastric)

#### Unusual symptoms

Unlike the more ‘everyday’ symptoms that participants could normalise, some symptoms represented a deviation from what was normal or acceptable for participants, and were thus regarded as unusual. Such bodily changes prompted participants to consider these a possible ‘symptom of something’. The labelling of symptoms as ‘unusual’ differed between participants. This was particularly noticeable for weight loss; some participants were concerned by the change in their normal physical state, while others viewed it in a positive way, even when the amount lost was considerable and often unexpected:

*'* *I’ve always had quite strong legs, because I’ve always exercised* [*…*] *I started to notice that my legs were getting thinner, and I just thought,* *"* *That’s strange.* *"* *Then my arms* [*…*] *I noticed I was starting to lose muscle content.* *'* (P22: male, aged 60–74 years, gastric)

*'* *I looked at myself in the mirror and it didn’t look like me, I was so slim, and this* *12* *–* *14* *jumper fit me absolutely perfect* [*…*] *I must have lost about two stone without even realising.* *'* (P02: female, aged ≥75 years, oesophageal)

Symptoms were also recognised as unusual when the experience did not match the participant’s understanding of how the symptom should present. Notably, half the oesophageal participants interviewed, and one gastric participant, described the unusual nature of their sickness, based on the symptom differing from what was expected when vomiting:

*'* *It weren’t a normal sick. To me, sick, it’s contents of your stomach, innit? What we call a* *"* *pavement pizza* *"* *but it weren’t like that.* *'* (P01: male, aged <60 years, oesophageal)

Instead of experiencing ‘normal’ vomiting, participants described bringing up *‘* *stringy goo* *’*, with the consistency likened to other bodily fluids such as spit or phlegm (Box 2). Many also commented on the quantity of fluid coming up and the difficulties experienced when trying to manage it:

Box 2 **Quotes illustrating unusual nature of vomiting**

- *'* *It was just like clear phlegm, just really weird, cos it was just clear; no food in it or anything.* *'* (P01: male, aged <60 years, oesophageal)
- *'* *It wasn’t so much the meat it was just like thick water, like wallpaper paste.* *'* (P04: male, aged <60 years, oesophageal)
- *'* *It was just this froth, like an enormous amount of spit.* *'* (P08: male, aged ≥75 years, oesophageal)
- *'* *I mean it used to be that thick at times I couldn’t get it out of my throat, I had to put my fingers in my mouth and get hold of it and drag it out.* *'* (P12: male, aged ≥75 years, oesophageal)
- *'* *That was like all stringy stuff, you know. Clear yeah. But you know there seemed yards and yards of it coming out.* *'* (P13: female, aged ≥75 years, oesophageal)
- *'* *Very quickly turning into not being able to eat or hardly drink without quite dramatic vomiting* *,* *which was primarily just stringy goo.* *'* (P17: male, aged 60–74 years, oesophageal)
- *'* *It’s this gooey phlegmy type thing that's almost elastic, it sort of stretches from my mouth into the toilet, if you know what I mean. It's odd stuff and it's obviously what the stomach produces, I think.* *'* (P18: male, aged ≥75 years, oesophageal)
- *'* *I had a lot of, well, I call it phlegm, but it wasn’t. It was very, very white and very clear, and with a lot of bubbles in it.* *'* (P25: male, aged 60–74 years, gastric)

*'* *Well like spit in my mouth* [*…*] *it just kept coming and that was sort of slimy, you know.* *'* (P13: female, aged ≥75 years, oesophageal)

*‘It used to be that thick at times I couldn’t get it out of my throat, I had to put my fingers in my mouth and get hold of it and drag it out* [*…*] *it could be six inches long sometimes.* *‘* (P12: male, aged ≥75 years, oesophageal)

This type of experience encouraged participants to consider seeking help as they were unable to find an explanation for their symptoms: *'I didn’t know what the devil it was'* (P18: male, aged ≥75 years, oesophageal).

#### Symptom progression and severity

Help-seeking was also considered when symptoms persisted over a period of time without resolving or worsened, increasing in frequency and/or intensity. This was particularly apparent for dysphagia:

*'* *Clearly it wasn’t going away and was although not getting* [*…*] *rapidly worse, clearly wasn’t getting any better and probably was getting worse.* *'* (P06: male, aged <60 years, oesophageal)

*'* *Things were starting to get worse, a lot worse, and then as I say, that day when I couldn’t drink the coffee I thought,* *"* *That’s it, I’ve had enough. I can’t carry on any longer* *".'* (P01: male, aged <60 years, oesophageal)

Others had the experience of a sudden symptom that felt overwhelming. This was often in relation to vomiting or severe pain, based on the shock of suddenly being sick or the intensity of the pain:

*'* *I had this overwhelming feeling of sickness and I thought,* *"* *I’m gonna be sick* *"* *,* *and I was actually sick, which shocked me ‘cause it just came out of the blue.* *'* (P05: female, aged 60–74 years, gastric)

*'* *I swallowed it and it was initially fine and then it wasn’t fine and I was just in absolute agony for half an hour, doubled over in agony, my tears were pouring out of my eyes because I felt so much pain.* *'* (P17: male, aged 60–74 years, oesophageal)

As symptoms progressed, participants found that daily activities were increasingly affected and their bodily changes became more apparent, to them and to those around them:

*'* *Sunday morning, I’m sitting in here,* [wife] *asked me to go and do something, and I couldn’t do it. I couldn’t go up the stairs. I got to the top of the stairs and I couldn’t breathe.* *'* (P19: male, aged 60–74 years, gastric)

### Help-seeking in primary care

Most participants (*n* = 112, 88%) sought help in primary care, having recognised the need for additional support in managing their symptoms: *'I thought “I can’t deal with it the way I am dealing with it so I need some help”.'* (P16: male, aged 60–74 years, gastric). This often followed an extended period of appraising and managing symptoms, with participants reporting a median patient interval of 79.5 days (range 1–712 days). Dysphagia was the most common symptom first presented (*n* = 44, 35%), although, as with the general experience of this symptom, this was driven almost entirely by those with oesophageal cancer (*n* = 43, 42%):

*'* *I just felt it go all the way down from top to bottom* [*…*] *you don’t normally feel the food go down, it just goes down, and this time I felt it go all the way down quite sharpish.* *'* (P11: male, aged 60–74 years, oesophageal)

**Figure 2.**
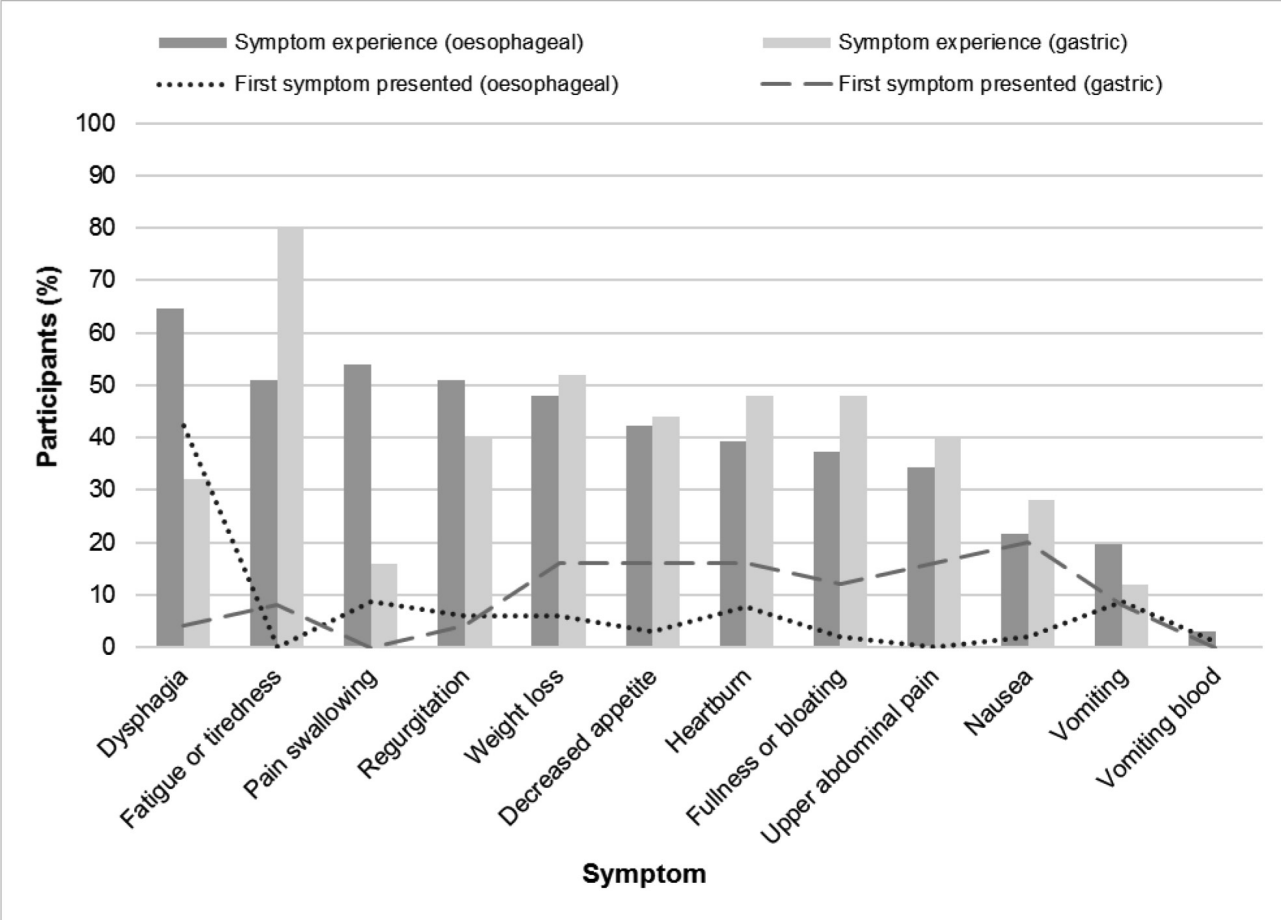
First symptom presented to the GP compared with symptom experience before diagnosis, stratified by cancer type.

Other symptoms were less frequently presented as the first symptom, despite their reported prevalence. For example, fatigue or tiredness was presented by only two gastric participants (8%), despite being the most common symptom for gastric participants before diagnosis (*n* = 20, 80%) and experienced by 72 (57%) participants across the cohort (Figure 2):

*'* *I said,* *"* *I’m absolutely worn out. I’ve got no strength. I can hardly walk. I just feel all in. I can't even get up the stairs, I’m out of breath, I’m puffing and panting. And I’m not like that* *".'* (P24: male, aged ≥75 years, gastric)

## Discussion

### Summary

This study used a survey and interviews to explore early symptom experience of oesophageal and gastric cancer from the perspective of newly diagnosed patients. Knowledge of these cancers was limited and symptoms were often ‘normalised’ or self-managed initially. Participants had multiple symptoms before diagnosis, with some variations in symptom experience between cancers. Despite heartburn being flagged by national campaigns as a key symptom, interviews highlighted how this term may not resonate with individuals. Participants often referred instead to reflux or indigestion when describing symptoms associated with heartburn, which may impact the interpretation of symptoms between patients and GPs. Those symptoms acknowledged as unusual, prolonged, or severe were more likely to be recognised as ‘symptom(s) of something’. As symptoms progressed, help-seeking was considered, with most participants consulting in primary care, although participants may not present all symptoms at the initial consultation, making it challenging for GPs to distinguish between benign disorders and suspected cancer.

### Strengths and limitations

The recruitment of 40% of eligible participants to the survey was a strong response rate considering the difficult time at which patients were approached, and is higher than the 19.5%–24% response rates observed in similar studies.^19–21^ Response to the interviews was positive with a broad range of participants recruited, allowing for in-depth exploration and comparison of experiences. A strength of the study design and analysis was that it combined survey and interview methods, and was guided by a conceptual framework, as recommended in early diagnosis research.^33^

The main limitation was the small sample size for survey analysis. Recruiting additional hospitals or sending questionnaire reminders could have increased participation; however, resources were limited for hospital recruitment and reminders may have caused patient distress at an already difficult time. Across the survey cohort, females, younger patients, and non-white ethnic groups were under-represented. These patients may have different experiences; therefore, generalisability of the results is limited. Patients with gastric cancer were also under-represented in the survey; however, almost half of the interview participants were diagnosed with gastric cancer, allowing in-depth exploration of their experiences and comparison with participants with oesophageal cancer. As the study recruited participants retrospectively, symptom reporting and narratives may have been subject to post hoc rationalisation; however, the data reflect reported participant experiences and perspectives.

### Comparison with existing literature

Existing literature is primarily based around quantitative methods, with the exception of a recently published study exploring symptom appraisal with 14 participants diagnosed with oesophageal cancer in Scotland (2012–2015).^38^ Interviews were conducted within 9 months of diagnosis, with the authors finding participants had limited knowledge of oesophageal cancer, initially appraising symptoms in the context of normal bodily functions until symptoms changed or worsened. This reflects findings in the current study, and resonates with research across other cancers, where subtle or intermittent symptoms are often not considered serious^25–27,39–41^ and initial bodily changes are attributed to benign causes.^39,42–45^ Another study explored the use of over-the-counter medication in Scotland with 25 patients diagnosed with lung (*n* = 14), colorectal (*n* = 4), or oesophago-gastric (*n* = 7) cancer in the preceding 12 months.^46^ More than half of the participants self-selected over-the-counter medications to initially manage symptoms, a finding similar to the current study, in which a third of participants used widely available medicines before consulting a healthcare professional. The findings identified a longer patient interval compared with previous studies (almost 3 months compared with up to 3 weeks),^5^ suggesting that current data may underestimate the period before diagnosis.

Previous studies using primary care medical records^47–49^ have explored symptoms as risk factors for oesophageal and gastric cancer. Stapley *et al* identified a 4.8% risk of cancer for patients with dysphagia and an increased risk for other symptom combinations such as epigastric pain with weight loss (4.2% risk).^47^ Dysphagia was primarily associated with oesophageal cancer, reflecting a previous study by Irving *et al* comparing the prevalence of this symptom between oesophageal and gastric cancer (77% versus 32%).^50^ The current findings also reflect this, with dysphagia primarily attributable to participants with oesophageal cancer. It is important to recognise other symptom combinations for gastric cancer patients, although the findings show that patients may not report all symptoms; therefore, risk models based on primary care records may not accurately reflect symptom experience. A novel finding from this study relates to the unusual sickness experienced by half of the interviewees with oesophageal cancer. Understanding the nature of symptoms, such as vomiting compared with *'* *stringy goo* *'*, may be important when assessing symptoms in primary care.

While symptom awareness campaigns are important for encouraging patients to seek help in primary care, the findings suggest that the key messages of the 2015 National Be Clear on Cancer campaign,^7^ focusing on frequent heartburn and dysphagia, may not have resonated with patients. As described, swallowing difficulties were rarely mentioned by gastric participants, suggesting that the campaign message may have failed to pinpoint relevant experiences for this group. In addition, only one-fifth of participants reported experiencing frequent heartburn and participants sometimes described symptoms of heartburn yet identified them as reflux or indigestion, making it difficult to relate to the campaign message. This has implications for primary care, where patients use the terms heartburn, reflux, or indigestion instead of describing their symptoms, as symptoms associated with these conditions may differ between patients.

### Implications for research and practice

Future research would benefit from investigating oesophageal and gastric cancers separately, to fully understand differences in symptom presentation. It is also important for future awareness campaigns to appreciate variations in symptom meanings and interpretation, to reduce potential misinterpretation of messages and symptom self-management where healthcare intervention would be more appropriate. Engaging with pharmacies and other providers of over-the-counter medication to utilise opportunities to encourage help-seeking could also prompt patients to consult sooner.

Patients often seek help following long periods of appraisal and may not present all relevant symptoms at their first consultation. Individual descriptions and terminology used by patients for upper gastrointestinal symptoms may hold different meanings to those understood by clinicians, increasing the possibility of misinterpretation or normalisation by the GP. This highlights the importance of careful exploration of the meaning of commonly used terms, such as reflux and indigestion, and ensuring that all relevant symptoms are identified during the consultation to avoid missed opportunities for earlier referral and diagnosis.

## References

[1] Cancer Research UK (2016). Oesophageal cancer incidence statistics. http://www.cancerresearchuk.org/health-professional/cancer-statistics/statistics-by-cancer-type/oesophageal-cancer/incidence.

[2] Cancer Research UK (2016). Stomach cancer incidence statistics. http://www.cancerresearchuk.org/health-professional/cancer-statistics/statistics-by-cancer-type/stomach-cancer/incidence.

[3] Cancer Research UK (2011). Oesophageal cancer statistics. http://www.cancerresearchuk.org/cancer-info/cancerstats/types/oesophagus/.

[4] Cancer Research UK (2011). Stomach cancer statistics. http://www.cancerresearchuk.org/cancer-info/cancerstats/types/stomach/.

[5] Lyratzopoulos G, Saunders CL, Abel GA (2015). The relative length of the patient and the primary care interval in patients with 28 common and rarer cancers. Br J Cancer.

[6] Department of Health (2011). Improving outcomes: a strategy for cancer. https://www.gov.uk/government/uploads/system/uploads/attachment_data/file/213785/dh_123394.pdf.

[7] Public Health England (2015). Be Clear on Cancer. http://www.nhs.uk/be-clear-on-cancer/oesophagogastric-cancer.

[8] Cook MB, Corley DA, Murray LJ (2014). Gastroesophageal reflux in relation to adenocarcinomas of the esophagus: a pooled analysis from the Barrett's and Esophageal Adenocarcinoma Consortium (BEACON). PLoS One.

[9] Spechler SJ, Jain SK, Tendler DA, Parker RA (2002). Racial differences in the frequency of symptoms and complications of gastro-oesophageal reflux disease. Aliment Pharmacol Ther.

[10] National Institute for Health and Care Excellence (2015). Suspected cancer: recognition and referral [Clinical Guideline CG12]. www.nice.org.uk/guidance/ng12.

[11] Javle M, Ailawadhi S, Yang GY (2006). Palliation of malignant dysphagia in esophageal cancer: a literature-based review. J Support Oncol.

[12] Adler DG, Baron TH (2001). Endoscopic palliation of malignant dysphagia. Mayo Clin Proc.

[13] Cancer Research UK (2014). Be Clear on Cancer: evaluation findings. http://www.cancerresearchuk.org/health-professional/early-diagnosis-activities/be-clear-on-cancer/oesophago-gastric-cancers-campaign/evaluation#BCOC_oesophago-gastric_evaluation.

[14] Koo S, Awadelkarim B, Dhar A (2017). The National Oesophagogastric Cancer Awareness Campaign: a locality outcome analysis from County Durham. Frontline Gastroenterol.

[15] Siau K, Yew AC, Hingley S (2017). The 2015 upper gastrointestinal "Be Clear on Cancer" campaign: its impact on gastroenterology services and malignant and premalignant diagnoses. Frontline Gastroenterol.

[16] Jones R (1995). Gastro-Oesophageal reflux disease in general practice. Scand J Gastroenterol.

[17] McColl E, Junghard O, Wiklund I, Revicki DA (2005). Assessing symptoms in gastroesophageal reflux disease: how well do clinicians' assessments agree with those of their patients?. Am J Gastroenterol.

[18] Lyratzopoulos G, Abel GA, McPhail S (2013). Measures of promptness of cancer diagnosis in primary care: secondary analysis of national audit data on patients with 18 common and rarer cancers. Br J Cancer.

[19] Walter FM, Rubin G, Bankhead C (2015). Symptoms and other factors associated with time to diagnosis and stage of lung cancer: a prospective cohort study. Br J Cancer.

[20] Walter FM, Emery JD, Mendonca S (2016). Symptoms and patient factors associated with longer time to diagnosis for colorectal cancer: results from a prospective cohort study. Br J Cancer.

[21] Walter FM, Mills K, Mendonça SC (2016). Symptoms and patient factors associated with diagnostic intervals for pancreatic cancer (symptom pancreatic study): a prospective cohort study. Lancet Gastroenterol Hepatol.

[22] Quality Health (2014). Cancer surveys. https://www.quality-health.co.uk/surveys/cancer-surveys.

[23] Gilhooly K, Green C, Richardson JTE (1996). Protocol analysis. Handbook of Qualitative Research Methods for Psychology and the Social Sciences.

[24] Ericsson KA, Ericsson KA, Charness N, Feltovich P, Hoffman RR (2006). Protocol analysis and expert thought: concurrent verbalizations of thinking during experts' performance on representative tasks. The Cambridge Handbook of Expertise and Expert Performance.

[25] Birt L, Hall N, Emery J (2014). Responding to symptoms suggestive of lung cancer: a qualitative interview study. BMJ Open Respir Res.

[26] Hall N, Birt L, Banks J (2015). Symptom appraisal and healthcare-seeking for symptoms suggestive of colorectal cancer: a qualitative study. BMJ Open.

[27] Mills K, Birt L, Emery JD (2017). Understanding symptom appraisal and help-seeking in people with symptoms suggestive of pancreatic cancer: a qualitative study. BMJ Open.

[28] Glasner T, van der Vaart W (2009). Applications of calendar instruments in social surveys: a review. Qual Quant.

[29] Mills K, Emery J, Cheung C (2014). A qualitative exploration of the use of calendar landmarking instruments in cancer symptom research. BMC Fam Pract.

[30] Braun V, Clarke V (2006). Using thematic analysis in psychology. Qual Res Psychol.

[31] Walter F, Webster A, Scott S, Emery J (2012). The Andersen model of total patient delay: a systematic review of its application in cancer diagnosis. J Health Serv Res Policy.

[32] Scott SE, Walter FM, Webster A (2013). The model of pathways to treatment: conceptualization and integration with existing theory. Br J Health Psychol.

[33] Weller D, Vedsted P, Rubin G (2012). The Aarhus Statement: improving design and reporting of studies on early cancer diagnosis. Br J Cancer.

[34] Pope C, Mays N, Popay J (2007). Synthesizing Qualitative and Quantitative Health Evidence: A Guide to Methods.

[35] Moayyedi P, Lacy BE, Andrews CN (2017). ACG and CAG clinical guideline: management of dyspepsia. Am J Gastroenterol.

[36] Vakil N, van Zanten SV, Kahrilas P (2006). The Montreal definition and classification of gastroesophageal reflux disease: a global evidence-based consensus. Am J Gastroenterol.

[37] Kahrilas PJ, Shaheen NJ, Vaezi MF (2008). American Gastroenterological Association medical position statement on the management of gastroesophageal reflux disease. Gastroenterology.

[38] Lewis L, Marcu A, Whitaker K, Maguire R (2018). Patient factors influencing symptom appraisal and subsequent adjustment to oesophageal cancer: a qualitative interview study. Eur J Cancer Care.

[39] Smith LK, Pope C, Botha JL (2005). Patients' help-seeking experiences and delay in cancer presentation: a qualitative synthesis. Lancet.

[40] Evans J, Chapple A, Salisbury H (2014). "It can't be very important because it comes and goes"— patients' accounts of intermittent symptoms preceding a pancreatic cancer diagnosis: a qualitative study. BMJ Open.

[41] Cook C, Brunton M, Pukepuke T, Tan AL (2018). Exploring communication during the journey from noticing bodily changes to a diagnosis of endometrial cancer. J Clin Nurs.

[42] Dobson C, Russell A, Brown S, Rubin G (2018). The role of social context in symptom appraisal and help-seeking among people with lung or colorectal symptoms: a qualitative interview study. Eur J Cancer Care.

[43] MacArtney J, Malmström M, Overgaard Nielsen T (2017). Patients' initial steps to cancer diagnosis in Denmark, England and Sweden: what can a qualitative, cross-country comparison of narrative interviews tell us about potentially modifiable factors?. BMJ Open.

[44] Molassiotis A, Wilson B, Brunton L, Chandler C (2010). Mapping patients' experiences from initial change in health to cancer diagnosis: a qualitative exploration of patient and system factors mediating this process. Eur J Cancer Care.

[45] Whitaker KL, Macleod U, Winstanley K (2015). Help seeking for cancer 'alarm' symptoms: a qualitative interview study of primary care patients in the UK. Br J Gen Pract.

[43] Notman F, Porteous T, Murchie P, Bond CM (2019). Do pharmacists contribute to patients' management of symptoms suggestive of cancer: a qualitative study. Int J Pharm Pract.

[47] Stapley S, Peters TJ, Neal RD (2013). The risk of oesophago-gastric cancer in symptomatic patients in primary care: a large case-control study using electronic records. Br J Cancer.

[48] Hippisley-Cox J, Coupland C (2013). Symptoms and risk factors to identify men with suspected cancer in primary care: derivation and validation of an algorithm. Br J Gen Pract.

[49] Hippisley-Cox J, Coupland C (2013). Symptoms and risk factors to identify women with suspected cancer in primary care: derivation and validation of an algorithm. Br J Gen Pract.

[50] Irving MJ, Lamb PJ, Irving RJ, Raimes SA (2002). Speeding up the diagnosis of oesophago-gastric cancer. Nurs Times.

